# Place Identity Strategies at University Constructed by Minority Arab-Israeli Student Groups

**DOI:** 10.3389/fpsyg.2021.665042

**Published:** 2021-07-13

**Authors:** Miriam Billig

**Affiliations:** Department of Sociology and Anthropology, Ariel University, Eastern R&D Center, Ariel, Israel

**Keywords:** place identity strategies, ethnic minority, ethno-political conflict, Arab-Israeli students, othering

## Abstract

The study examines the place identity of minority group Arab-Israeli students studying at a campus affiliated with the Israeli hegemonic majority, against the backdrop of the enduring Israeli–Palestinian conflict. The study analyzes place identity construction strategies utilized by these students, and the formation patterns of the new place identity reflected in everyday campus life. Subjective experiences of students were revealed through the ethnographic and qualitative phenomenological methodology and in-depth interviews. From the findings, it became apparent that life under conditions of ongoing ethnic–political conflict forces minority groups to develop strategies regarding their place identity. These strategies are fluidly employed depending on the specific context of time and place. Four place identity strategies were identified: overt, borrowed, avoidant, and ideological. Key factors contributing to the construction of each strategy were discovered: rooted place identity; gender expectations, and proactive or passive attitude to place. Implementation tactics such as individual versus collective approaches, distancing from other groups, and the flow between multiple identities were also uncovered. The study asserts that the strategies, tactics, and key factors revealed in the research contribute to place identity theory and will enrich other place identity studies of minority groups and communities in fluid contexts. Expanding theoretical discourse with respect to the strategies and tactics of place identity could promote the opportunity for integration and coexistence.

## Introduction

This study describes the construction of place identity by Arab-Israeli university students coping with their status as members of an ethnic minority in the hegemonic Jewish majority. In ethnic and national terms, Palestinian-Arab citizens of Israel (from here on Arab-Israeli) are part of the Palestinian nation but also account for close to 20% of Israel’s population (Hager and Jabareen, 2016; Shafir, 2018). Approximately 90% of Arab-Israelis reside in homogeneous Arab villages or cities; approximately 10% reside in mixed Jewish and Arab cities (Shafir, 2018). Encounters between Jews and Arabs occur in the workplace and in leisure venues. Residential separation is present in social and cultural settings (Shdema et al., 2020) and in exclusion and discrimination grounded in the Israeli–Palestinian ethno-national conflict (Hager and Jabareen, 2016). The public Arab education system is conducted in Arabic, in separate environments. The civic identity of young Arabs in Israel is consequently shaped with little or no interactions with the Jewish majority (Abu-Rabia-Queder, 2014).

The intractable Israeli–Palestinian conflict is expressed in extreme fear, tension, and a common sense of victimhood, which have become part of the collective memory of both sides of the conflict (Bar-Tal, 2001, 2007; Bar-Tal et al., 2009; Hadar, 2019). Peace-building efforts in recent decades have reduced prejudices and reinforced more positive perceptions of the Other (Oren and Bar-Tal, 2014), and the conflict has become primarily reflected in a political context rather than the discourse of everyday interpersonal interactions. These changes largely reflect multiculturalism, bilingualism, and a sense of shared destiny (Smooha, 2013).

This shift is aptly illustrated by the increasing number of Arab students enrolled in Israeli universities and their increasing percentage in higher education in Israel (Council of Higher Education (CHE), 2019). For some, universities are the first opportunity to connect with Jews and the first social framework in which they experience being labeled a minority. Mutual prejudices, language barriers, and cultural differences challenge these young students and lead them to develop various coping strategies (Abu-Rabia-Queder, 2008; Arar, 2017). Like other students around the world, Arab students undergo a period of adjustment in their transition to university. The move to a new geographic location and the start of student life entail place identity formation and the deconstruction and rebuilding of identity (Chow and Healey, 2008; Holton and Riley, 2016).

This study seeks to examine the process of place identity construction by minority group Arab-Israeli students during their studies on an Israeli campus. It identifies formation patterns of the new place identity and how these patterns are reflected in everyday campus life, and explores the factors affecting this process.

### Theory

Individual attitudes to specific geographic places are the focus of a broad multidisciplinary field of research that encompasses cultural, sociological, and psychological—cognitive and emotional—dimensions. Geography affects how individuals attach to and identify with a place, how they ascribe it meaning and values, and how narratives that constitute building blocks of their selfhood are built. The most widely used concepts in literature describing this are place attachment and place identity; the distinction between them, however, is not always well defined (Dixon and Durrheim, 2000; Lewicka, 2008, 2014; Devine-Wright, 2009; Billig and Lebovitz, 2014).

Pinkster (2016) distinguishes usage of the term “place identity” as the “nature or identity of a particular place” from its environmental psychology usage to describe the contribution a place makes to self-identity. Peng et al. (2020) differentiated between “people’s place identity” and “place identity of a place”—a term frequently used in geography. This study considers place identity a cognitive structure that represents memories, emotions, beliefs, and meanings related to a specific place, through which individuals shape and interpret their identity. Place identity develops with reference to a place of which the individual has a direct, concrete experience, and which the individual perceives as having subjective and symbolic significance (Proshansky et al., 1983; Lalli, 1992).

Place identity differs from other social identities that are not associated with belonging or attributing meaning to a specific place (Belanche et al., 2017). Personal and social identities might be understood as different levels of self and social categorization. Social identity relates to self-perception of the individual in terms of shared similarities with others, while distinguishing between “us”—in-group, and “them”—out-group (Turner et al., 1994). A physical place may be seen as a component of self-categorization producing a sense of belonging to specific environments, and an element of personal identity. These environments may also be meaningful for a group; they may therefore be a social category (Valera et al., 1998) and a component of social identity (Vidal et al., 2012). For example, Lalli (1992) suggested a person’s urban identity reflects their connection to their own city as a geographic place and to its people, and their disassociation from the residents of other cities (Belanche et al., 2017). The complex fabric of one’s relationship with a place includes relations with the local community and their social status in it, which in turn affects the construction of place identity (Hummon, 1992; Billig, 2013). Place identity can develop through the residence, through affiliation with a local community, or through the construction of a regional identity (Cuba and Hummon, 1993).

Place identity is an active, dynamic construction which evolves as the individuals interact with the environment, and engage with the stories they tell themselves about the place, its history, and its people (Manzo, 2005; Lewicka, 2008). It may preserve continuity or discontinuity when the individual chooses, or is forced, to relocate, and facilitates the development of self-distinctiveness, self-esteem, and self-efficacy (Twigger-Ross and Uzzell, 1996).

The literature mostly refers to place identity formation as an individual experience, but it is sometimes observed collectively (Kuusisto-Arponen, 2009; Knez and Eliasson, 2017; Knez et al., 2018). National or ethnic identity may alter memories related to a place and ascribe to them different interpretations, creating the foundation for the development of distinct collective place identities (Wheeler, 2017).

Discursive negotiations on the “definition of a place” (Dixon and Durrheim, 2000) may entail exclusion from the group or stipulate acceptance of a specific meaning as a condition of group membership. On the other hand, a new collective and common place identity may be developed by members of different groups based on a shared perception of the local environment. This may bridge social rifts and transcend conflicting ideologies. The “student villages” in Israel, set up by civil society organizations, may illustrate this point. These student groups, from different and conflicting social and political backgrounds, sharing a common social agenda to help underprivileged local communities, formed collective place identities and came together through a sense of group loyalty and pride, an “Esperit de Corps” (Billig and Lebovitz, 2014).

As noted earlier, the concept of place attachment is used to describe an individual’s attitude to a specific geographic place (Billig, 2006). One dimension of place attachment is a sense of rootedness, which evolves through continuous residence in one place (McAndrew, 1998). Hummon (1992) distinguished between the everyday rootedness of people who rarely leave their hometown, which they take for granted, and the ideological rootedness of those who consciously select their place of residence. He noted place identity is strong in both cases. Following Hummon, Lewicka (2011, 2014) distinguished traditional place attachment of those displaying everyday-rootedness related to memories, from the active place attachment of those displaying ideological rootedness developed when immigrants connect to the roots of a place by demonstrating an active interest in its history.

The mobility characterizing contemporary society (Cresswell, 2006) entails dynamic place attachment that is reformulated and open to change whenever an individual moves through “the fixities and flows” of life and multiple places of significance (Di Masso et al., 2019). Accordingly, the concept of place identity was extended in more recent literature to include non-residential places, including imagined places, of major significance through which the individual moves. By this interpretation, place identities can be considered assemblages that generate a sense of anchoring and rootedness in multiple, varying places (Manzo, 2005; Cresswell, 2014; Di Masso et al., 2019).

Students’ lives reflect such mobility and dynamism simply because of relocation to a remote university town. Frequently, students feel varying degrees of rootedness and place attachment to their hometown (McAndrew, 1998). The social and geographic distance from home evokes mixed feelings, occasionally including alienation, but some students may gradually develop a sense of home and belonging and a new place identity which joins their rooted place identity (Chow and Healey, 2008; Billig and Lebovitz, 2014). In their study of British students, Holton and Riley (2016) stressed the importance accommodation plays in forming a sense of home and student identity. Qingjiu and Maliki (2013) demonstrated how campus life contributed to Malaysian students’ development of place attachment and place identity while noting national students formed a stronger place identity compared to international students. Vidal et al. (2010) showed an association between residential mobility and place identity of students in Barcelona. Israeli students developed a stronger place identity when they lived longer in major cities vs. small or medium-sized cities (Casakin et al., 2015).

A study conducted in Israel showed that rooted place identity referring to the hometowns (places of permanent residence) of Arab-Israeli students is stronger than the rooted place identity of Jewish students. The Arab-Israeli students attributed their place identity to Arab neighborhoods, towns, and regions, although their exposure to Jewish students weakened it (Shdema et al., 2020). Arar (2017) showed that interaction and open discussion among Arab and Jewish students on campus, and the Arab students’ growing awareness of marginality and exclusion during their education, contribute to the re-construction of their academic identity.

A process of renegotiation of Israeli identity has emerged in recent years, including expressions of Jewish Israelis’ increasing willingness to include Arab-Israelis in their in-group (Hadar, 2019). Arab-Israelis undergo corresponding changes. As members of a society in transition from traditionalism to modernity, and as an ethno-national minority, Arabs in Israel have complex identities (Shdema et al., 2020). The dominant dichotomous Israeli/Palestinian and Jewish/Arab discourse appears to have become increasingly inclusive and less polarized between “Palestinization” and “Israelization” (Amara and Schnell, 2004; Smooha, 2013). Arab-Israelis also allow themselves greater fluidity when defining their identities (Ross and Razon, 2015); bilingualism and combined use of Hebrew and Arabic may demonstrate this (Dubiner, 2018).

Many young Arabs have, to varying degrees, a sense of being torn between their Palestinian identity and national belonging, and their civic identity as Israelis. Some consequently form a hyphenated self-identity that is both separate and common (Sirin and Fine, 2007; Hammack, 2010). This study explores the formation of place identity by young Arab-Israeli students in a society that is subject to an ongoing conflict. Transition of students to university constitutes a case study for place identity formation that is not based on traditional place attachment or on longstanding residence, personal ties, and memories. Instead, place identity development at university is based on encounters with a new place and new people and reflects how students see the university as gradually forming part of their identity during their studies (Chow and Healey, 2008; Arar, 2017).

Literature discusses the place identity formation of immigrants and ethnic minorities (Main, 2013; Trąbka, 2019), and competition between rival groups over the meaning attributed to a place (Dixon and Durrheim, 2000; Di Masso et al., 2011). Researchers have addressed place identity in societies that are or were divided by war, such as the Balkans or Northern Ireland (Hopkins and Dixon, 2006; Lewicka, 2008), or by political conflict as in Barcelona (Di Masso et al., 2011). Marzorati (2013) examined the relationship between established Italian community members of a Milan neighborhood and immigrants using the neighborhood park. She described an interplay of place identity construction and a process of othering by the established community in their attitude to the immigrants. *Othering* here is understood as the casting of an outsider or a group in the role of “the other”—the action of making a clear contrast between “us” and “them,” the “in-group” and the “out-group” (Turner et al., 1994). These studies explored the connection between collective identity formation and the meanings of place. While Shdema et al. (2020) examined changes in the traditional rooted place identity of Arab-Israeli students with respect to their home city, this study examines strategies of new place identity formation among Arab-Israeli students moving to university.

### Aim of the Study

The study examines students from a minority group living under enduring ethnic–political conflict, who chose to study at a campus affiliated by the hegemonic majority. The study aims to (a) examine the process of place identity construction by the minority group during their studies on campus, and their ongoing encounters with other students; (b) identify the formation patterns of the new place identity, discover whether this process involves fixed patterns or diverse ones, and demonstrate how these patterns are reflected in everyday campus life; (c) explore factors affecting the new place identity formation after students relocate to university, and during frequent movements between hometown and campus; and (d) recommend operative actions to assist students in these situations.

## Materials and Methods

This ethnographic study offers a reflection of the world of Arab-Israeli university students through their life stories and their definitions and interpretations of place on campus. The study focuses on how attitudes of students toward the campus environment are affected by their perceptions of the campus as a site requiring a redefinition of their identity. It uses a qualitative phenomenological methodology to reveal the subjective experiences of students and examine the meanings they ascribe to the place (Smith, 2003).

### Study Population

The study was conducted in a Jewish Israeli university located 30 min from Tel Aviv and 50 min from Jerusalem. Arab students account for 10% of the university’s student body of 17,000. All Arab students are Israeli citizens and are either Muslim (Sunni), Christian, or Druze. Most Arab students live in student dormitories or rent accommodation in the adjacent city. The rest travel to and from home every day.

### Sample

Thirty-five Arab students were selected for the study using the snowball sampling method. Each interviewee was asked to recommend potential interviewees from among fellow Arab students with opinions, approaches, or lifestyles different from theirs. All interviewees were Muslim or Christian Arab-Israeli citizens; some were from the Bedouin sector (Bedouins are a tribal, originally nomad, ethnic group of Muslim Arabs). Interviewees comprised 18 females and 17 males aged between 18 and 24. Close to half (46%) originally lived in the Triangle area (an area in central Israel populated mainly by Arab citizens), 51.5% in the Galilee, and 2.8% in western Jerusalem; 20% were Bedouins who completed military service. Approximately 29.7% grew up in families with four or more children, while the remainder grew up in families of three children or fewer. The majority (78%) reported having a father who was full-time employed, and 41% reported having a mother who worked outside the home.

Interviewees studied a diverse range of disciplines: Most male students studied engineering, computer sciences, and political science, while most female students studied nursing, behavioral sciences, and sociology.

### Instruments and Procedure

Semi-structured, in-depth interviews were conducted at a location selected to be convenient to interviewees: the campus cafeteria, an outdoor location on campus, or the office of researchers. Interviews lasted between 60 and 120 min and were recorded and transcribed by the interviewer. The interviewer was the author of this study.

Interviewees were invited to freely and without interruption describe their personal and collective experiences, and their feelings and opinions about the university. They were asked whether their experiences and opinions represented personal attitudes only, or those of other Arab students. Interviewees were also questioned about their daily life on campus, including interactions with Jewish and Arab students. Particular attention was paid to their treatment from the university and other students, their sense of belonging on campus and pride in their place of education. Students gave consent to participate and spoke candidly during the interview. Several said they enjoyed the interview feeling it was important to make their voice heard.

The interviews were analyzed using the grounded theory method (Smith, 2003). Analysis was carried out in four stages: Interview transcriptions were carefully read and divided into meaningful units; the strategies students adopted, either consciously or unconsciously, were identified to build a hierarchy; main themes were identified and explanations developed. A theoretical model was produced adding further weight to the existing theory.

### Reliability

Actions taken to strengthen the reliability of the study included maintaining the chain of evidence—all collected data were preserved, from the first step of research to the conclusions to ensure accurate analysis (Smith, 2018; Yin, 2018); gaining the interviewees’ trust in a sensitive and careful pre-interview conversation by the interviewer; adding the research assistant as a second judge of the ethnographic texts to ensure impartiality. The research assistant of a separate age group, gender, and with a different life outlook was asked to approve or reject each of the strategies identified by the interviewer. This is an inductive ethnographic study, which includes few theoretical premises; although the interviewer and research assistant had significant room for their subjective interpretations (Geertz, 1973), at the end of the process a consensus regarding all four strategies was achieved.

### Ethics Approval Statement

Students were guaranteed complete anonymity, and names and hometowns were changed. The university ethics committee approved this study.

## Results

**FIGURE 1:**
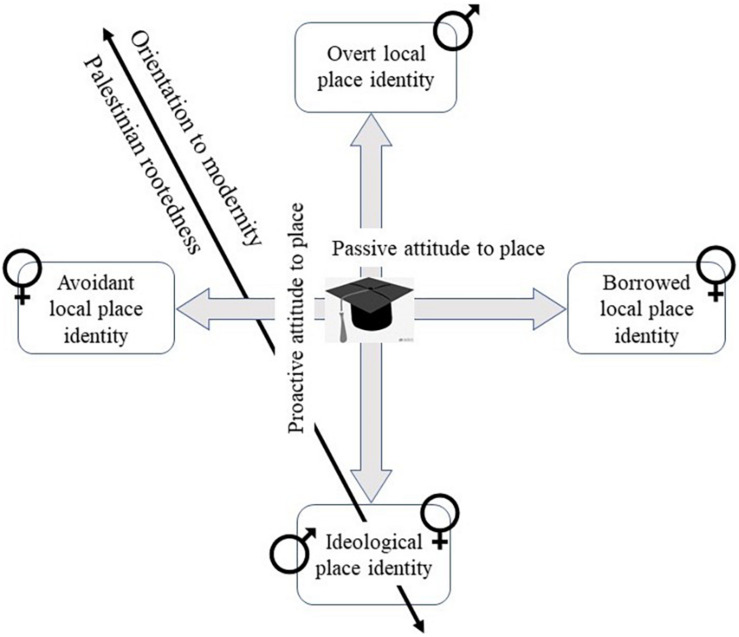
Strategy formation of place identity on campus.

Four place identity strategies adopted by the Arab students were identified (see Figure 1, model), each with advantages and disadvantages:

### Overt Local Place Identity

“Being part of the majority group”

This strategy was used mainly by male Bedouin Muslim Arab students who enrolled in the university after completing military service (IDF, Israel Defense Force). Their rooted place identity was “Israeli” and included a sense of belonging and commitment to the state and to their extended family (the *hamula*). As university students, they adopted a campus-based place identity, and like many Jewish students, several were employed on campus in security-related jobs. In many respects, they expected their on-campus experience to be a continuation of their military service during which they felt equal to their Jewish peers in the IDF. Their experience on campus did not, however, live up to these expectations.

As guards on campus, their adopted place identity included a sense of responsibility for the place and a desire to fit into the campus social life. This strategy included the “adoption” of biographic and behavioral elements similar to those of their Jewish peers, including styles of attire and speech (using Hebrew). They expressed pride having served in the army, in their family’s ties with Jews, and in their political affiliation with Israel’s mainstream politics. They differentiated themselves from Arab students who did not serve in the military. Basel, a representative of this group, stated:

We are different from the other Muslim Arabs […] very loyal to the state […] I met on campus religious Jewish students who never before spoken to one not Jewish… it didn’t frighten me…I shared my experience from the army with them… I didn’t stay aloof.

Basel followed his cousin and his friends from the police force, in enrolling at the university. His interview revealed he and his friends paid a price for their overt local place identity. Basel describes the resulting sense of dual alienation:

I saw how the Muslim guys, consider me like a “traitor” and scorn when they randomly meet as if to say “he’s not one of us” … and some of the Jews are also restrained … and that’s something that I never felt in my military service … some look at me strangely when I speak in Arabic…

Basel is critical of the other Muslim Arab students on campus, and stresses the differences between them and him:

There are some guys who never spoke to a Jew before in their life … they are constantly preoccupied with Palestine … being alone among themselves … opposing Israel … I know that they don’t like the fact that I hang around with Jews or that I went to military service … it’s a really old dispute between the Bedouins and the other Muslims.

However, the Bedouin students revealed that their academic performance is lower than the Jewish student average, and most of them are required to attend tutorials offered to Arab students on campus. They were surprised to learn about the glass ceiling which prevented escape from their minority marginal status, as Basel clarifies:

At my village, I’ve always been considered an excellent student and during my army service I was a good soldier who won appreciation and respect from the Israelis … At university I was surprised to discover how hard the studies were, that my level of Hebrew and English was not good enough, and that I needed help to pass my exams. It was a shock for me … It reduced my belief in my ability to equal Jewish students’ achievements.

Furthermore, being identified on campus as “part of the group of Arabs” repeatedly undermined their well-defined Israeli identity and its attached equal status.

A sense of confusion also arises from the description given by Riyad, another Bedouin army graduate. He described a negative experience with a Jewish guard at the campus gate: “That guard’s look, staring at me…as if I were there to commit a terrorist attack.”

Despite the academic challenges that undermined Basel’s self-image as an outstanding student in his home village, he continued to reaffirm a place identity that belonged to and was integrated into Jewish mainstream life in Israel. Actively developing social ties with Jewish students was one of the methods he used to affirm his place identity. The new place identity affords students such as Basel a “place of refuge” from their home village and occasionally their close family, which are increasingly viewed as old-fashioned, rigid, and conservative. These students consider their efforts to shed the shackles of tradition, albeit temporarily, and to adopt more modern and permissive behaviors, as a symbol of their Israeli-modern identity. Hamoudi, a peer of Basel, described the process:

In my village there’s nothing to do, and everyone knows you, and you can’t make any move without someone talking about you […] I am like everyone else … I can have fun … It’s fun being in a new place … it’s fun to take a little break from my family […] I’m here ‘living the life.’

Although the everyday rootedness (Hummon, 1992) and traditional place attachment of students such as Basel and Hamoudi are anchored to their village and tribe, their work as guards and other activities on campus allow them to develop an active place attachment (Lewicka, 2008) and a new, overt local place identity. The interview materials indicate that despite unanticipated academic challenges, their encounters with Arab students who did not serve in the army reinforces their sense of othering, differentiating “us” who served in the army from “them” who did not (Marzorati, 2013; Roberts and Schiavenato, 2017). Bedouin students attribute to themselves a higher status than other Arab students on campus, a result of their engagement with Jewish Israeli society. They seem to consider their assimilation into Jewish society on campus an inevitable challenge and view the university as a space of opportunity to reaffirm their fluid Israeli identity (Ross and Razon, 2015). It is also seen as a lever to a future career in the Israeli job market.

### Borrowed Local Place Identity

“Playing this game is exhausting”

This strategy characterizes female students who choose to conceal their Arab identity on campus and outwardly project a borrowed, yet passive, Jewish–Israeli identity. This strategy was adopted by students from liberal modern families who typically maintained ties with Jews. Several of these students had attended a Jewish high school. Their place identity functions as a disguise. They appear to identify with the university, enjoy campus life, and consider it to be an element of their identity. However, they recognize the temporary, borrowed, and limited nature of this place identity. These students noted they are able to fit in with Jews as long as the latter were unaware they were Arabs. On discovery of their Arab identity, the relationship became more ambivalent for both parties.

Students with a borrowed place identity rely on their personal and cultural capital: They speak fluent Hebrew with no accent, and with similar attire and behavior, they fit in with the majority culture on campus. They do not express any political or ideological views, and the strategy they adopt is mainly for the sake of convenience and utility. They are conscious of their instrumental use of place identity, which helps them integrate, and is adopted with the aim of achieving maximum success both in their studies and in their student social lives.

Nadia, a Christian Arab from East Jerusalem, attended a Jewish high school in the western part of the city and spoke fluent Hebrew. She had dyed golden-blond hair (uncharacteristic to Arab-Israeli women), wore jeans and branded T-shirts, and concealed her Arab identity. Her name is common among Jewish females. Without disclosing that she had come from an Arab neighborhood in eastern Jerusalem, she was careful to note she was born in the city. This daily strategy of disguise helps Nadia connect to male and female Jewish students; she makes no effort to disabuse them of their assumption that she is also a Jew. Still, when a terrorist attack was committed by a Palestinian perpetrator, she found it difficult to continue her pretense:

…I started to get close to my Jewish friends on campus. I used their lesson summaries and spent the breaks with them. Truly, it was nice with them. And then the terrorist attack occurred … a woman was murdered in Jerusalem. The students started to curse the Palestinian who committed the attack, and then one sunk to making racist remarks about Arabs in general. It was a difficult moment for me, I am sure that if she had known that I was an Arab, she would have been more careful … Suddenly I really came face to face with what they really think of me, of us…

A borrowed local identity places these female students as comparative observers critical of the relations between the Jewish majority and Arab minority. They are also critical of the other Arab students’ preoccupation with politics and history. Alice, a Muslim student from the Galilee, believes she represents a new generation of young people who are pragmatic and individualist:

Today’s young people are focused on their future success … so they sing the Palestinian anthem outwardly while inside they are happy that they are Israeli … people should understand that it’s convenient for them to live in a strong, modern, well-organized country.

Nicole, a Muslim female student from the Galilee, describes the price of her borrowed place identity—a sense of impermanence and lack of connection, accompanied by stress and mental load:

I’m not ashamed of my Arab identity but I don’t feel comfortable emphasizing it at the university, and I have to remember it all the time […] I always thought of myself as an Arab who gets along well with Jews … but this game is exhausting… […] The outcome is that I don’t have any friends among the Arab students. I also know that after graduation, all my ties with the Jews will not last … I’m waiting to get out of here…

Their borrowed place identity forces these students to re-examine the price of their Israeli identity and redefine the meaning of their Arab identity. Their daily efforts to disguise their Arab appearance paradoxically brings them closer to their Arab roots, although this remains incomplete and hyphenated (Sirin and Fine, 2007; Hammack, 2010). In many respects, their campus experience seems to mirror the experiences of migrants who move between their home and destination countries (Lewicka, 2011). Indeed, impermanence and disconnectedness characterize many people in post-modern society (Manzo, 2005; Di Masso et al., 2019). However, the Israeli–Palestinian conflict, which is constantly present in the background (Bar-Tal, 2007), and typically moves to center screen upon an extreme event such as a terrorist attack, creates a unique situation that directly affects these female students. When racist remarks percolate through social conversations, they are forced to deal with their borrowed place identity and shift back to their roots.

### Avoidant Local Place Identity

“Here today, gone tomorrow”

This strategy characterizes female Arab students from a traditional Muslim background who enrolled at university despite family objections to their studying outside their village. It reflects the lack of support from family and community. They mitigate conflicts on campus by exclusively viewing it as a site of study. They avoid social interactions with other students, either Arab or Jewish. Although enrolling at the university was their choice, they recognize the threat being on campus creates to their reputation in their home village. Concerned that inappropriate behavior might damage prospects of a good match, they are careful to take precautions to reduce this risk as far as possible.

The place identity of these students on campus, as a consequence, is weak and avoidant. They leave their rooms only to attend classes, and they speak only with their roommates or few acquaintances, and refrain from joining social groups, even those attended by other female Arabs. They are careful to wear traditional attire, including the hijab, and are sure to leave no imprint on the campus, appearing unaffected by it. This place identity adopted through the reluctance of students to identify with a new place expresses and preserves their rooted place identity.

On campus, they conduct themselves as “present but absent.” Maisa’s account represents many female students like her. She enrolled at the university to earn a certificate in health sciences from a prestigious institution. She chose to follow her cousin in studying at the university despite her parents’ objections to it being far from home. Maisa stressed it was important to prove she could be trusted to protect her reputation, which is why she decided to live in the dorms with a roommate from her village. She chose to maintain a “low profile” on campus and return home as frequently as possible. Her encounters with other Jewish and Arab students reinforced her decision to keep her distance from them: “I don’t hang out with Arab students … if someone from the village will see me—he might talk…”

Maisa and her friend Amal dress modestly and wear the hijab. Their religious attire stands out on campus and to some extent contradicts their desire to reduce their presence in the space. Their conduct is more careful than it would be in their village, and they remain in the classroom during breaks. Lucy explained that by doing this they can avoid “unpleasant” looks from Jews on campus, especially during security or national events. Amal described the heavy personal toll she paid, in the form of the loneliness and the sense of non-belonging that she experiences:

Like many female Arab students, I am pretty alone here … we don’t think it’s appropriate for girls to walk around on campus […] I may have said only some sentences to Jews the whole year, […] I feel that I don’t belong … I wait for the weekends so I can go home.

Students like Maisa and Amal remain at the margins of their classes, and their mastery of the Hebrew language is low. Noor states that she has a difficult time with her studies and does not always understand her course assignments, but she does not ask her instructors questions and refuses assistance. She is “unaware if other students share lesson summaries.” She admits her academic achievements are poor, but as long as she earns a diploma at the end of the program, maintaining her good reputation is her priority.

The strong rooted place identity held by these young women (Billig and Lebovitz, 2014) is anchored in the conservative patriarchal parental home (Chow and Healey, 2008). An avoidant place identity strategy allows them to maintain their home identity while justifying their involvement in university studies and residence on campus. They do, however, pay a price in the form of loneliness and lessen their chances of academic success. In many cases, the formation of a place identity entails self-affiliation with the local community (Lalli, 1992; Cuba and Hummon, 1993; Billig, 2013), something these women intentionally avoid.

### Ideological Place Identity

“Ultimately, it’s us or them”

The fourth place identity strategy is a proactive-ideological local identity, which characterizes the majority of male Arab students and a minority of female Arab students on campus. Differences in interpretation of this identity were found: Male students deliberately stand out on campus through their unique attire and hairstyles; their emphasized loud speech in Arabic; and their constant presence in groups at specific locations on campus. They adopt an activist isolationist Arab-Palestinian identity and are engaged in campaigns to create change. Female students wear jeans and modest blouses, similar to those they wear in their village. They do not conceal their Arab identity but instead proclaim it with pride.

The primary motivation of this strategy was a need for social ties with students from a similar background in the new, foreign environment. Manal added another motivation for adopting it—being at the center of social influence and power. She explained: “I stick to my principles, I came to create a change, and social support is important to me …. I admit that I have become closer to feminism…”

The group Manal joined frequently meets during breaks, after classes, and in the evenings. They occasionally go to a restaurant off campus. During all of these meetings, they engage in frank conversations and share experiences. Group members help one other with their studies, share lesson summaries translated into Arabic, and exchange information on courses and instructors. Their social cohesion facilitated the formation of a collective identity as members of an Arab minority, as Abed described:

Suddenly you discover that you know that person from that village and another person from another village …. You understand the differences between the regions […] but you also see how similar we are … in contrast to the Jews … in the songs we know … in our family structure … in what’s acceptable and what’s not…

The place identity formed by these students reflects covert, and occasionally overt, isolationism, as Mahmoud noted: “I have my friends and that’s enough.” However, according to Manal and Ranya, female students came to campus better prepared than their male counterparts. They had met Jews through parental connections or at work, and they spoke fluent Hebrew learnt in high school. Manal has never concealed her identity but felt campus life challenged her to demonstrate pride in her Arab identity:

Some people are confused and think that I am a Jew, I immediately correct them. Some people see that I am an Arab and they recoil …. Those, I put them in their place, too.

When students with an ideological place identity gather at their chosen sites on campus, they appropriate these locations as their own separate “Arab”’ spaces. While this strengthens their self-confidence, they are aware their isolationism may disconcert others. Yakin recounts:

It’s important for everyone to know that Arabs live here!. I think that people are even afraid to walk near us … We don’t feel part of the campus … Sometimes people look at us like “What are they doing there all the time, sitting among themselves … maybe they’re planning a terrorist attack?” No less […] In the end, it’s a Jewish campus and we’re Arabs…

The political consciousness they develop during free time spills over into class and is reflected in a transition to activism. For example, Camal described a case in class when the professor mentioned the Arab world with a condescending attitude; he felt he could not keep quiet and must present the other side by reminding them of how many people were killed in the bombings of Gaza. Similarly, Hadde described expressing her opinions to her Jewish friends in class:

The professor claimed that we don’t have [gender] equality, that it’s difficult to be an Arab. That made me very angry. I stood up and replied: “That’s not true!! It all depends on your family!” … I saw the professor look at me, deliberating how to respond because I don’t look like an Arab woman. So I said, “Yes, I’m an Arab! And my parents pushed me to study in this university and not in a college close to home, even though my father is religious.”

These students’ ideological activist place identity is manifest in political action on campus and even involvement in nation-level political action. They join efforts to produce a change in various spheres on campus, such as demands for Arabic signage, traditional Arab music and dancing on Student Day, a prayer room for Muslim students, and observance of the month of Ramadan. Ranya decided to volunteer with the Student Association after realizing that while Arab men had a student representative, there was no equivalent for Arab women. She adopted a “soft” activist approach to promote change.

For these students, ideological place identity strategy transforms the university into a site of maturation, a space for developing social connections and political consciousness. Yet the university also frames them as an ethno-national minority that is only partially integrated into the Israeli public sphere. Nevertheless, their active ideological place identity may change during visits to their hometown—their rooted place identity at the village may become passive; there is a flow between identities. Male students typically form a collective ideological place identity, emphasizing othering by distancing themselves from other groups, especially the Jewish majority (Marzorati, 2013; Roberts and Schiavenato, 2017). The meaning they attribute to the campus (Wheeler, 2017) is anchored in their Arab-Palestinian identity (Arar, 2017). In contrast, without othering, female students adopt a softer activism strategy that facilitates self-realization and promotion of self-interests, as strategy that includes encounters and contact with the Jewish majority. Their ideological place identity is occasionally reflected in feminist activism (Abu-Rabia-Queder, 2014; Abu Oksa Daoud, 2017; Sa’ar et al., 2017).

The four place identities identified in the descriptions of the Arab-Israeli students (see Figure 1) are represented on two axes. The first axis represents an activist place strategy, which ranges from overt to covert ideology and was adopted by the majority of the male students. The second axis represents a passive place strategy adopted only by female students, which includes borrowed vs. avoidant identity. The majority of female interviewees favor integration on campus and embracing modernity, while the minority tended to reinforce their Palestinian rootedness by preserving their home identity (avoidant identity, male ideological identity).

## Discussion

The experience of Arab-Israeli students choosing Jewish Israeli universities was impacted by the Israeli–Palestinian conflict—one of several factors to influence the formation of student place identity. To cope with life under this ethno-national conflict, Arab students formed four contrasting place identity strategies representing four corresponding student subgroups. The development of these strategies was a complex process involving several components; strategies were fluidly employed depending on the specific context of time and place. The conflict was reflected to varying degrees in these strategies. Two groups chose an activist approach: One group supported integration into the Jewish majority on campus, adopting overt place identity strategy; the other, wishing to retain segregation from the Jewish majority, chose a contrasting strategy of ideological place identity. The remaining two groups favored a passive approach: The first adopted a borrowed identity strategy with the aim of provisionally assimilating into the Jewish majority; the second eschewed integration through avoidant place identity.

Three factors affected the formation of these strategies: The rooted place identity formed at home while growing up, and held by the students on arrival; gender expectations; and interactions with separate student groups on campus set against the backdrop of the intractable conflict. It appears strategy choice was greatly influenced by a rooted place identity which had been affected by previous experience meeting Jews. Differences were found between the overt strategy adopted by Bedouin students who had served in the military, and the ideological strategy adopted by those who had not. These differences were largely related to Bedouin identification with the Jewish majority (Zeedan, 2019); this contrasted the ideological group’s distrust of the hegemonic majority driven by support for the Arab minority’s struggle for rights.

Female students from traditional patriarchal homes (Abu Oksa Daoud, 2017) adopted an avoidant place identity strategy, those from liberal homes adopted a borrowed local strategy, and those with strong feminist awareness developed an activist-ideological place identity (Sa’ar et al., 2017). Gender also affected student’s cognitive-affective experiences on campus and the features of social interactions they developed. While male students were inclined toward activism, most female students had a more passive approach, although a small group was identified as choosing a feminist activist ideological strategy.

All four strategy groups to some degree reflected everyday rootedness (Hummon, 1992), and deep traditional place attachment to their hometown and environment where they grew up (Lewicka, 2008, 2011). These students, however, chose different approaches toward modernization and human mobility. Males choosing overt local place identity expressed Israeli identity, identification with the state of Israel, and favored modernity. Male students who chose an ideological place identity strategy had more traditional beliefs and opinions and tended toward “Palestinization” (Smooha, 2013). Female students adopting an avoidant place identity strategy were rooted and traditional, opposing modernity and Israeli culture. Those adopting an ideological or borrowed place identity leaned more strongly toward “Israelization” and modernity; they were more likely to embrace mobility and flow (Di Masso et al., 2019).

Like other minority students across the world (Chow and Healey, 2008; Holton and Riley, 2016), Arab students’ encounters on campus led to the construction of place identity strategies based on intergroup relations and sometimes othering of other groups (Marzorati, 2013; Roberts and Schiavenato, 2017). These encounters led to redefinition not only of their place identities but also of personal and social identities they formed later in life (Turner et al., 1994). The process of their identity formation was influenced by the renegotiation of their national-Palestinian identity and their civic-Israeli identity (Arar, 2017). Meeting other Arabs and Jews on campus, for some, catalyzed a reinforcement of their Palestinian identity. For others, on-campus encounters offered an opportunity to strengthen the Israeli component of their identity (Shdema et al., 2020). This study highlights that Arab students cannot be merely “students” free from apparent ethnic identity. Analysis of the four strategies underscores the challenge of aligning with a soft, neutral Israeli identity not based on ethnic or religious affiliation and disconnected from the Jewish–Arab conflict.

Students mostly sought out other like-minded peers forming a supportive community for their chosen strategy. In this respect, these findings reinforce the argument that place identity can form not only through attachment to a place, but also through community affiliation and the building of a fabric of community relations (Cuba and Hummon, 1993; Billig, 2013). Females who developed a borrowed place identity strategy and chose to distance themselves from their own culture while on campus, were exceptions. This strategy served them as long as the issue of the Israeli–Palestinian conflict did not emerge in discourse that may have revealed their identity and compelled them to take a public stand on the issue (Bar-Tal, 2007).

Place identity, in literature, is generally viewed as an individual phenomenon. Collective tactics, however, exist in varying degrees in the strategies this study identifies (Kuusisto-Arponen, 2009; Knez and Eliasson, 2017). Borrowed place identity reflects separate individual coping, yet reverts to authentic collective affiliation when a political event occurs. Avoidant strategy entails minimal connections, using an individualist tactic limited only to friends from the same village; overt local identity depends on the presence of similar Bedouin students; and ideological strategy is a collective place identity clearly characterized by movement on campus in groups separated from other Jewish and Arab students. Students who choose this last strategy may, through its activism and prominence, find themselves able to join a formative group celebrating its uniqueness. This cohort continues to maintain internal and external discourses, about social status, similar and conflicting values, political narratives, and different interpretations of life on campus (Twigger-Ross and Uzzell, 1996; Dixon and Durrheim, 2000). Intergroup connection had a positive influence on the students who chose avoidant, overt, and ideological place identities. It helped them adjust to the new environment and empowered their self efficacy and self-esteem by allowing inclusion in a group of people like them. Unlike these groups, students who chose the borrowed place identity preferred connecting with majority Jewish students to improve their sense of self-esteem, and in this way, they could improve their university achievements (Twigger-Ross and Uzzell, 1996; Billig, 2013).

## Conclusion

An individual’s relationship to physical space is a multidisciplinary research field that explores different aspects of how one develops a connection to and identification with a geographical place. This study reveals that life under conditions of ongoing ethno-political conflict forces minority groups to construct strategies regarding their place identity. The study expands the context of place identity to include extreme situations, and identifies key factors affecting the formation of each strategy: rooted place identity; gender expectations; and proactive or passive attitude to place. Careful observation enables the identification of implementation tactics such as individual versus collective approaches, and the flow between rooted and campus place identities depending on time and place. The study asserts that the strategies, tactics, and key factors revealed contribute to place identity theory and will enrich other place identity studies of minority groups and communities in fluid contexts.

These insights potentially contribute to the design of campus environments better adapted to the needs of various student groups, reflecting greater sensitivity to their on-campus, non-academic needs. Using insights gained in this study, universities can promote integration opportunities on campus for interested minority groups. Students’ perceptions of the campus and its communities affect their behaviors within the environment: They may serve as guards at entrance gates, thus gaining visibility; they may move in groups and meet in fixed places; they may assimilate with the Jewish majority; or they may shun encounters by self-isolating in dorms. Such behaviors affect their perception by other Arab and Jewish students and reinforce the process by which place becomes part of their self-identity.

This research has limitations which may be resolved in follow-up studies. Although all interviewees agreed to participate, several may have hesitated to criticize the university in the presence of Jewish interviewers. A follow-up study should therefore use Arab-Israeli researchers, and interviews should be conducted in Arabic. A larger sample and a longer study design that covers students’ university years may validate the strategies identified here. This study focused on the subjective perceptions of Arab students and therefore primarily represents their perspectives on university life. Future studies may explore how the majority is affected by place identities of minority groups.

Place identities formed at university affect the identity of students in their professional and personal lives after graduation, and their identity as Arab citizens in Israel. Understanding the need of minority students to repeatedly redefine themselves based on location, job, and strategy may help efforts to build day-to-day peace between rival groups. The distinction between place identities in intractable political, racial, or ethno-national conflicts may help create a space for each minority group and promote opportunities for integration and peaceful coexistence.

## Data Availability Statement

The original contributions presented in the study are included in the article/supplementary material, further inquiries can be directed to the corresponding author.

## Ethics Statement

The studies involving human participants were reviewed and approved by Ariel University. The patients/participants provided their written informed consent to participate in this study.

## Author Contributions

The author confirms being the sole contributor of this work and has approved it for publication.

## Conflict of Interest

The author declares that the research was conducted in the absence of any commercial or financial relationships that could be construed as a potential conflict of interest.
